# Revisiting Clinical Trials Using EGFR Inhibitor-Based Regimens in Patients with Advanced Non-Small Cell Lung Cancer: A Retrospective Analysis of an MD Anderson Cancer Center Phase I Population

**DOI:** 10.18632/oncotarget.1028

**Published:** 2013-06-04

**Authors:** Jennifer Wheler, Gerald Falchook, Apostolia M. Tsimberidou, David Hong, Aung Naing, Sarina Piha-Paul, Su S. Chen, John Heymach, Siqing Fu, Bettzy Stephen, Jansina Y. Fok, Filip Janku, Razelle Kurzrock

**Affiliations:** ^1^ Department of Investigational Cancer Therapeutics – a Phase I Clinical Trials Program, The University of Texas MD Anderson Cancer Center, Texas; ^2^ Department of Hematopathology, The University of Texas MD Anderson Cancer Center, Texas; ^3^ Department of Thoracic/Head and Neck Medical Oncology, The University of Texas MD Anderson Cancer Center, Texas; ^4^ Moores Cancer Center, University of California, San Diego

**Keywords:** EGFR mutation, EGFR wild-type, non-small cell lung cancer, phase I trials, resistance, squamous cell

## Abstract

**Purpose:**

Single-agent EGFR inhibitor therapy is effective mainly in patients with lung cancer and *EGFR* mutations. Treating patients who develop resistance, or who are insensitive from the outset, often because of resistant mutations, other aberrations or the lack of an *EGFR* mutation, probably requires rational combinations. We therefore investigated the outcome of EGFR inhibitor-based combination regimens in patients with heavily-pretreated non-small cell lung cancer (NSCLC) referred to a Phase I Clinic.

**Methods:**

We reviewed the electronic records of patients with NSCLC treated with an EGFR inhibitor-based combination regimen: erlotinib and cetuximab; erlotinib, cetuximab and bevacizumab; erlotinib and dasatinib; erlotinib and bortezomib; or cetuximab and sirolimus.

**Results:**

*EGFR* mutations were detected in 16% of patients (21/131). EGFR inhibitor-based combination regimens were administered to 15 patients with *EGFR*-mutant NSCLC and 24 with *EGFR* wild-type disease. Stable disease (SD) ≥6 months/partial remission (PR) was attained in 20% of *EGFR*-mutant patients (3/15; two with sensitive mutations and secondary resistance to prior erlotinib, and one with a resistant mutation), as well as 26% of evaluable patients (5/19) with wild-type disease. One of three evaluable patients with squamous cell histology achieved SD for 26.5 months (*EGFR* wild-type, *TP53*-mutant, regimen=erlotinib, cetuximab and bevacizumab).

**Conclusions:**

Eight of 34 evaluable patients (24%) with advanced, refractory NSCLC evaluable for response achieved SD ≥6 months/PR (PR=3; SD ≥6 months=5) on EGFR inhibitor-based combination regimens (erlotinib, cetuximab; erlotinib, cetuximab and bevacizumab; and, erlotinib, bortezomib), including patients with secondary resistance to single-agent EGFR inhibitors, resistant mutations, wild-type disease, and, squamous histology.

## INTRODUCTION

Activation of the epidermal growth factor receptor (EGFR) signaling pathway is known to play a significant role in the pathophysiology of non-small cell lung cancer (NSCLC)[1]. It may also be important in other tumors[2]. Aberrant activation of the signaling pathway may occur due to mutations in exons 18 through 21, which encode part of the tyrosine kinase domain and are bundled around the ATP-binding pocket of the enzyme[1-4]. There is a broad literature on the efficacy of EGFR inhibitors in NSCLC[3-6] . Although many patients with *EGFR*-mutant NSCLC respond to EGFR inhibitors initially, they eventually develop resistance to treatment. Therefore, combination approaches to overcome resistance is an area of active clinical research[7, 8].

The role of EGFR inhibition in patients with wild-type *EGFR* and lung cancer has been debated. Studies with erlotinib show increased survival in unselected patients with lung cancer,[9] though there is a general consensus that patients with sensitive *EGFR* mutations are most likely to benefit[3, 4]. Recently, preclinical studies have demonstrated that EGFR can signal via a kinase-independent pathway[10], suggesting a role for combining EGFR kinase inhibitors and antibodies. Furthermore, preclinical models suggest that several molecules synergize with EGFR inhibitors, including the multikinase inhibitor dasatinib[11] and the proteasome inhibitor bortezomib[12]. Herein, we report our experience with EGFR-based combination regimens in patients with advanced, heavily-pretreated NSCLC referred to a phase I clinic, including those with secondary resistance to erlotinib, resistant mutations, and *EGFR* wild-type disease.

## RESULTS

### EGFR mutations

Twenty-one of 131 NSCLC patients (16%) tested had *EGFR* mutations. Twenty-five *EGFR* mutations were present in those 21 individuals. Four patients had two *EGFR* mutations. Ten of the 25 *EGFR* mutations were present in exon 19; three in exon 20; and, 12 in exon 21. Of the four patients who had two *EGFR* mutations, three of them had two *EGFR* mutations in exon 21 and 1 patient had an *EGFR* mutation in exon 19 and exon 20. Deletions in exon 19 (n = 9) and the L858R substitution mutation in exon 21 (n = 7) were the two most common types of mutations.

### Treatment

Fifteen of the 21 patients (71%) with an underlying *EGFR* mutation were enrolled in five clinical trials that included an EGFR inhibitor combination (Patients and Methods and Table 2).

Of the remaining six *EGFR*-mutant NSCLC patients, one patient was treated on a clinical trial that did not include an EGFR inhibitor, one patient was referred to hospice, one patient died soon after being seen, and three patients were treated with single-agent erlotinib by their primary oncologist or on study.

### Patients treated with EGFR inhibitor-based combinations

Patient characteristics of the 15 *EGFR* mutation-positive and 24 *EGFR* wild-type NSCLC patients treated with EGFR inhibitor-based combination regimens are summarized in Table 1.

**Table 1:** Baseline characteristics of 15 evaluable patients with *EGFR* mutation-positive NSCLC and 24 patients with *EGFR* wild-type NSCLC treated with EGFR inhibitor-based combination regimens

Variable	EGFR-mutant n=15	EGFR wild-type n=24
Sex, n (%)		
Male	8 (53)	14 (58)
Female	7 (47)	10 (42)
Age (years)		
Median	65	69
Range	29-76	42-82
Ethnicity, n (%)		
Caucasian	6 (40)	21 (88)
Asian	5 (33)	0 (0)
Hispanic	2 (13)	2 (8)
African American	2 (13)	1 (4)
Histology, n (%)		
Adenocarcinoma	13 (87)	20 (83)
Squamous cell	1 (7)	3 (13)
Adenosquamous	1 (7)	0 (0)
Neuroendocrine	0 (0)	1 (4)
*EGFR* mutation, n (%)		
Exon 19	6 (40)	0 (0)
Exon 20	2 (13)	0 (0)
Exon 21	4 (27)	0 (0)
Two mutations	3 (20)	0 (0)
*KRAS* mutation, n (%)		
Positive	0 (0)	2 (8)
Negative	13 (87)	18 (75)
Unknown	2 (13)	4 (17)
*PIK3CA* mutation, n (%)		
Positive	2 (13)	2 (8)
Negative	5 (33)	11 (46)
Unknown	8 (53)	11 (46)
History of smoking, n (%)		
Ex-smoker	7 (47)	16 (67)
Never smoked	8 (53)	8 (33)
Number of prior therapies		
Median	4	2
Range	0-7	1-7
Previous EGFR inhibitors, n (%)		
Yes	12 (80)	8 (33)
No	3 (20)	16 (67)
ECOG PS		
0	4 (27)	5 (21)
1	10 (67)	14 (58)
2	1 (7)	5 (21)

Abbreviations: ECOG, Eastern Cooperative Oncology Group; *EGFR*, Epidermal Growth Factor Receptor; *KRAS*, V-Ki-ras2 Kirsten rat sarcoma viral oncogene homolog; NSCLC, Non-Small Cell Lung Cancer; PS, Performance status; *PIK3CA*, Phosphatidylinositol 3-kinase, catalytic, alpha polypeptide; PR, Partial response; SD, Stable disease

### Co-existing mutations in 15 *EGFR*-mutant patients treated with EGFR-based regimens

Simultaneous mutations in other genes were assessed when tissue was available. Two of seven *EGFR* mutation-positive patients (29%) assessed had a *PIK3CA* mutation. One patient (case #15, Table 2) had an E545K mutation in exon 9 of the *PIK3CA* gene in addition to the *EGFR* mutation (T847I in exon 21; unknown sensitivity to EGFR inhibitors). A second patient (case #5, Table 2) had an E542K mutation in exon 9 of the *PIK3CA* gene in addition to two known sensitive *EGFR* mutations (L858R and G873E) in exon 21. No patient that underwent treatment with an EGFR inhibitor-based combination had a *KRAS* mutation (though one patient who was not treated had a G12C mutation in addition to a resistant *EGFR* [D761N] mutation in exon 19).

**Table 2:** Characteristics of 15 patients with *EGFR* mutations^§^ treated with EGFR inhibitor-based regimens

Case No.	Histology	*EGFR* mutations (exon)	Sensitive/Resistant†	Concomitant mutations	Previous EGFR inhibitor therapy			Treatment in phase I program		
					Yes/ No	Best response	TTF (months)	EGFR inhibitor	Best response	TTF (months)
1	Adenocarcinoma	insertion/ deletion in exon 19	Sensitive	*PIK3CA*: Not done* KRAS: No *TP53*: Not done	Yes	PR	24.8	erlotinib, dasatinib	PD**	2
2	Adenocarcinoma	insertion in exon 20	Resistant	*PIK3CA*: No *KRAS*: No *TP53*: Not done	No	NA	NA	erlotinib, cetuximab	PR	13+
3	Adenocarcinoma	deletion in exon 19	Sensitive	*PIK3CA*:Not done *KRAS*: No *TP53*: Not done	Yes	SD	12.0	erlotinib, cetuximab, bevacizumab	SD	4
4	Adenocarcinoma	deletion in exon 19	Sensitive	*PIK3CA*:Not done *KRAS*: No *TP53*: Not done	Yes	PR	26.1	erlotinib, cetuximab	PD**	1
5	Adenocarcinoma	L858R (exon 21), G873E (exon 21)	Sensitive, Sensitive	*PIK3CA*: E542K (exon 9) *KRAS*: No *TP53*: Not done*	Yes	SD	14.3	erlotinib, cetuximab, bevacizumab	PR	9+
6	Adenocarcinoma	D830Y (exon 21), V834A (exon 21)	Unknown significance Unknown significance	*PIK3CA*:No *KRAS*: No *TP53*: Not done	Yes	SD	4.0	cetuximab, sirolimus	SD	4
7	Adenocarcinoma	L858R (exon 21)	Sensitive	*PIK3CA*:No *KRAS*: No *TP53*: Not done	Yes	PR	8.1	erlotinib, bortezomib	SD	2
8	Adenocarcinoma	T790M (exon 20), deletion in exon 19	Resistant, Sensitive	*PIK3CA*: Not done* KRAS: No *TP53*: Not done	Yes	PR	1.9	erlotinib, cetuximab	PD**	2
9	Adenocarcinoma	L858R (exon 21)	Sensitive	*PIK3CA*:No *KRAS*: No *TP53*: Not done	Yes	SD	11.5	erlotinib, dasatinib	PD	2
10	Adenocarcinoma	L858R (exon 21)	Sensitive	*PIK3CA*:Not done *KRAS*: No *TP53*: Not done	Yes	PR	5.5	erlotinib, bortezomib	SD	10+
11	Adenocarcinoma	insertion in exon 20	Resistant	*PIK3CA*: Not done* KRAS: No *TP53*: Not done	No	NA	NA	erlotinib, cetuximab, bevacizumab	SD	3
12	Adenocarcinoma	deletion in exon 19	Sensitive	*PIK3CA*:No *KRAS*: No *TP53*: Not done	Yes	SD	3.5	cetuximab, sirolimus	SD	4
13	Adenosquamous	deletion in exon 19	Sensitive	*PIK3CA*: Not done* KRAS: Not done* TP53: Not done*	Yes	PR	3.4	erlotinib, cetuximab	PD	1
14	Adenocarcinoma	deletion in exon 19	Sensitive	*PIK3CA*:Not done *KRAS*: Not done *TP53*: Not done	Yes	SD	8.0	erlotinib, cetuximab	PD**	2
15	Squamous cell carcinoma	T847I (exon 21)	Unknown significance	*PIK3CA*: E545K (exon 9) *KRAS*: No *TP53*: Not done*	No	NA	NA	erlotinib, dasatinib	PD**	0.4

Abbreviations: *EGFR*, Epidermal Growth Factor Receptor; *KRAS*, V-Ki-ras2 Kirsten rat sarcoma viral oncogene homolog; NA, Not applicable; NSCLC, Non-Small Cell Lung Cancer; PR, Partial response; PIK3CA, Phosphatidylinositol 3-kinase, catalytic, alpha polypeptide; PD, Progressive disease; SD, Stable disease; TTF, Time to treatment failure; TP 53, Tumor Protein p53

§ Of the remaining six EGFR-mutant NSCLC patients who were not treated on EGFR inhibitor-based regimens, two patients had a sensitive deletion in exon 19, one patient had a resistant D761N mutation in exon 19, one patient had 2 EGFR-sensitive mutations, L858R and L833V, in exon 21, and two patients had a sensitive L858R mutation in exon 21

† Sensitive or resistant is denoted based on survey of the literature.

* not done because tissue was not available for molecular analysis

** clinical progression/new metastasis

+ did not progress at the time of analysis

### Other mutations in *EGFR* wild-type patients treated with EGFR-based regimens

Two of 13 patients (15%) with *EGFR* wild-type disease assessed for *PIK3CA* mutation had an E545K mutation in exon 9 of the *PIK3CA* gene (cases #15 and 23, Table 3). Two of 20 patients (10%) with EGFR wild-type evaluated for *KRAS* mutation had a G12D mutation (cases #20 and 21, Table 3). Of the two patients with *EGFR* wild-type disease evaluated for *TP53* mutation, one had an R196 mutation in exon 6 (case #1, Table 3) and the other had a V157F mutation in exon 5 (case #19, Table 3).

**Table 3:** Characteristics of 24 NSCLC patients with EGFR wild-type disease treated with EGFR inhibitor-based regimens

Case Number	Histology	Other mutations	Previous EGFR inhibitor therapy			Treatment in phase I program		
			Yes/No	Best response	TTF (months)	EGFR inhibitor-based therapy	Best response	TTF (months)
1	Squamous cell carcinoma	*PIK3CA*: No *KRAS*: No *TP53*: R196 (exon 6)	No	NA	NA	erlotinib, cetuximab, bevacizumab	SD	26.5
2	Adenocarcinoma	*PIK3CA*: Not done *KRAS*: No *TP53*: Not done	No	NA	NA	erlotinib, cetuximab, bevacizumab	PD**	2.0
3	Adenocarcinoma	*PIK3CA*: Not done *KRAS*: Not done *TP53*: Not done	No	NA	NA	erlotinib, cetuximab, bevacizumab	PD**	0.9
4	Adenocarcinoma	*PIK3CA*: Not done* KRAS: No *TP53*: Not done	Yes	PD	2.7	erlotinib, cetuximab, bevacizumab	SD	4.1
5	Adenocarcinoma	*PIK3CA*: Not done *KRAS*: No *TP53*: Not done	No	NA	NA	erlotinib, cetuximab, bevacizumab	SD	4.4
6	Adenocarcinoma	*PIK3CA*: No *KRAS*: No *TP53*: Not done	Yes	SD	6.2	erlotinib, cetuximab, bevacizumab	SD	9.2
7	Adenocarcinoma	*PIK3CA*: Not done *KRAS*: No *TP53*: Not done	No	NA	NA	erlotinib, cetuximab, bevacizumab	PD	2.0
8	Adenocarcinoma	*PIK3CA*: Not done *KRAS*: No *TP53*: Not done	No	NA	NA	erlotinib, cetuximab, bevacizumab	PD**	3.8
9	Adenocarcinoma	*PIK3CA*: No *KRAS*: No *TP53*: Not done	No	NA	NA	erlotinib, cetuximab, bevacizumab	SD	6.5
10	Adenocarcinoma	*PIK3CA*: No *KRAS*: No *TP53*: Not done	No	NA	NA	erlotinib, cetuximab, bevacizumab	SD	11.0
11	Adenocarcinoma	*PIK3CA*: Not done *KRAS*: No *TP53*: Not done	No	NA	NA	erlotinib, cetuximab, bevacizumab	SD	2.2
12	Adenocarcinoma	*PIK3CA*: Not done* KRAS: No *TP53*: Not done	No	NA	NA	erlotinib, cetuximab, bevacizumab	PR	4.1
13	Adenocarcinoma	*PIK3CA*: No *KRAS*: No *TP53*: Not done	No	NA	NA	erlotinib, cetuximab, bevacizumab	SD	3.3
14	Squamous cell carcinoma	*PIK3CA*: Not done *KRAS*: No *TP53*: Not done	No	NA	NA	erlotinib, cetuximab, bevacizumab	PD	2.1
15	Adenocarcinoma	*PIK3CA*: E545K (exon 9) *KRAS*: No *TP53*: Not done*	No	NA	NA	erlotinib, cetuximab, bevacizumab	SD	3.1+
16	Adenocarcinoma	*PIK3CA*: Not done *KRAS*: No *TP53*: Not done	No	NA	NA	erlotinib, cetuximab	SD	2.0
17	Squamous cell carcinoma	*PIK3CA*: No *KRAS*: Not done* TP53: Not done	Yes	SD	6.0	erlotinib, cetuximab	too early	1.5+
18	Adenocarcinoma	*PIK3CA*: No *KRAS*: No *TP53*: Not done	Yes	PD**	0.7	erlotinib, cetuximab	too early	1.6+
19	Adenocarcinoma	*PIK3CA*: No *KRAS*: No *TP53*: V157F (exon 5)	Yes	PD**	0.9	erlotinib, cetuximab	PD**	0.5
20	Adenocarcinoma	*PIK3CA*: No *KRAS*: G12D *TP53*: Not done*	Yes	PD	2.3	erlotinib, cetuximab	too early	0.0+
21	Adenocarcinoma	*PIK3CA*: No *KRAS*: G12D *TP53*: Not done*	No	NA	NA	erlotinib, cetuximab	too early	0.1+
22	Adenocarcinoma	*PIK3CA*: No *KRAS*: Not done *TP53*: Not done	Yes	PD**	0.8	erlotinib, bortezomib	PD**	0.3
23	Neuroendocrine	*PIK3CA*: E545K (exon 9) *KRAS*: Not done *TP53*: Not done	No	NA	NA	erlotinib, dasatinib	SD	2.8
24	Adenocarcinoma	*PIK3CA*: Not done *KRAS*: No *TP53*: Not done	Yes	PD	2.0	cetuximab, sirolimus	too early	0.9+

Abbreviations: *EGFR*, Epidermal Growth Factor Receptor; *KRAS*, V-Ki-ras2 Kirsten rat sarcoma viral oncogene homolog; NA, Not applicable; NSCLC, Non-Small Cell Lung Cancer; PR, Partial response; *PIK3CA*, Phosphatidylinositol 3-kinase, catalytic, alpha polypeptide; SD, Stable disease; PD, Progressive disease; TTF, Time to treatment failure; *TP 53*, Tumor Protein p53

* not done because tissue was not available for molecular analysis

** clinical progression/new metastasis

+ did not progress at the time of analysis

### Responses to EGFR inhibitor-based combinations in *EGFR*-mutant NSCLC patients

**Figure 1:**
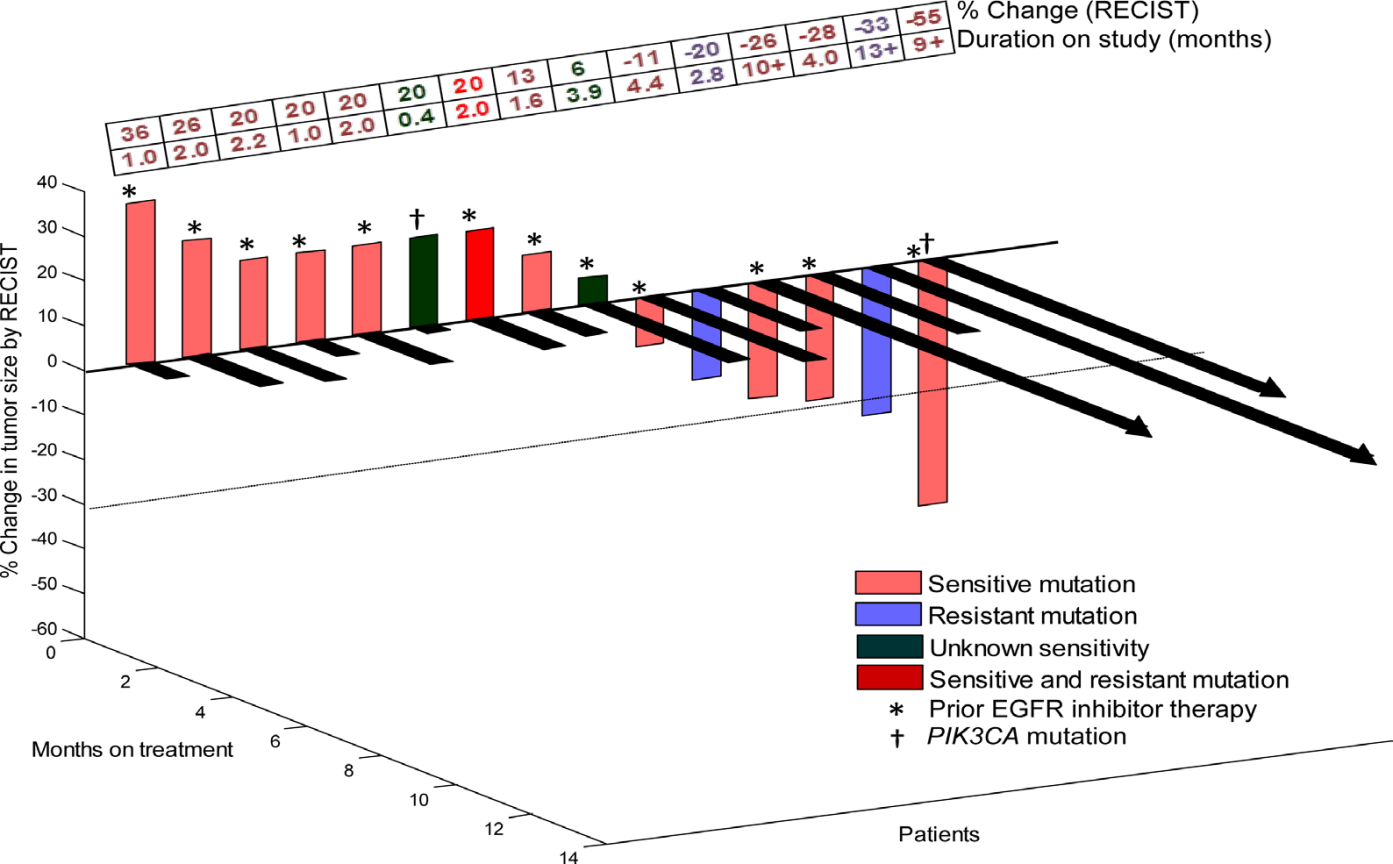
3-D Waterfall plot Best response by RECIST, of 15 NSCLC patients with *EGFR* positive-mutations treated with an EGFR inhibitor-based regimen. Patients with clinical progression or with new metastases were graphed as 20% progression. Time to treatment failure in months is represented by solid lines and the arrow indicates that the patient was still on study when the data was censored. Patients with *PIK3CA* mutations in addition to *EGFR* mutation and patients who received prior EGFR inhibitor therapy are designated as such. Dotted horizontal line at -30% indicates border for partial response.

Three of 15 evaluable (20%) patients attained either PR (n=2; cases #2 and 5, Table 2) or SD ≥6 months (n=1; case #10, Table 2). Six patients came off study prior to post-treatment imaging evaluation due to clinical progression (all of whom were arbitrarily graphed as 20% progression in Figure 1). One patient (case #2, Table 2) achieved a PR (33% decrease; duration=13+ months) on erlotinib/cetuximab despite having a known *EGFR*-resistant mutation (insertion in exon 20)[13]. This patient had previously received two lines of standard chemotherapy but had not received prior EGFR inhibitor therapy. TTF on the last standard treatment before referral to phase I was 2.0 months. A second patient (case #5, Table 2) with two known *EGFR*-sensitive mutations (L858R and G873E in exon 21) and a *PIK3CA* mutation (E542K in exon 9) had a PR (55% decrease; duration=9+ months) on erlotinib/cetuximab/bevacizumab. This patient had received six lines of prior therapy including single-agent erlotinib (TTF=14.3 months). TTF on the last standard treatment before referral was 4.5 months. A third patient (case #10, Table 2) with a known *EGFR*-sensitive mutation (L858R) in exon 21 attained SD for 10+ months on erlotinib/bortezomib. This patient had received six lines of prior therapy including single-agent erlotinib (TTF = 5.5 months). TTF on the last standard treatment before referral was 5.9 months.

**Figure 2:**
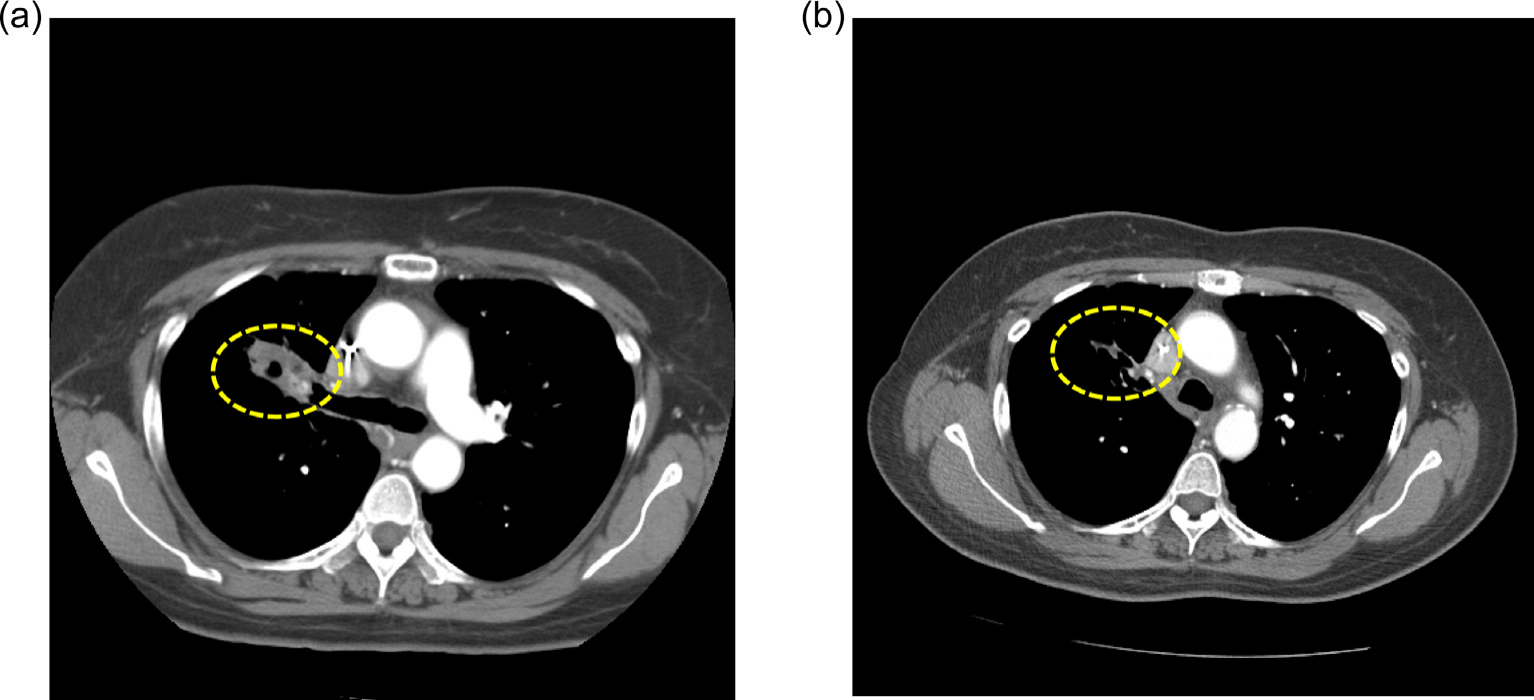
Computed tomography (CT) scans of a NSCLC patient (case #5, Table 2) with two sensitive *EGFR* mutations (L858R in exon 21 and G873E in exon 21) and a *PIK3CA* mutation (E542K in exon 9) a) CT at baseline, and b) CT taken 5 months after treatment initiation with erlotinib/cetuximab/bevacizumab demonstrating a PR (-55%). Duration of response = 9+ months. Patient had received prior erlotinib for 14.3 months.

### Responses and TTF in *EGFR*-mutant patients who had received prior EGFR inhibitors

Of the 12 patients who had progressed previously on EGFR inhibitors and received an EGFR inhibitor-based combination regimen after referral, two patients (17%) achieved either PR (n=1; case #5, Table 2; duration=9+ months) or SD≥6 months (n=1; case #10, Table 2; duration=10+ months) on this study. Of the three patients who achieved SD ≥6 months/PR on this study, two patients (67%; cases #10 and #5) had received prior erlotinib therapy as single-agent and had progressed. The TTF on the EGFR inhibitor-based combination therapies on this study is ongoing at 10+ and 9+ months vs. 5.5 and 14.3 months respectively on prior single-agent erlotinib. For the 12 *EGFR*-mutant patients who received prior EGFR inhibitors, median TTF on their EGFR inhibitor-based combination regimen after referral was 2 months as compared to 8 months on a prior EGFR inhibitor (p=0.044)

### Responses to EGFR inhibitor-based combinations in NSCLC patients with *EGFR* wild-type disease

**Figure 3:**
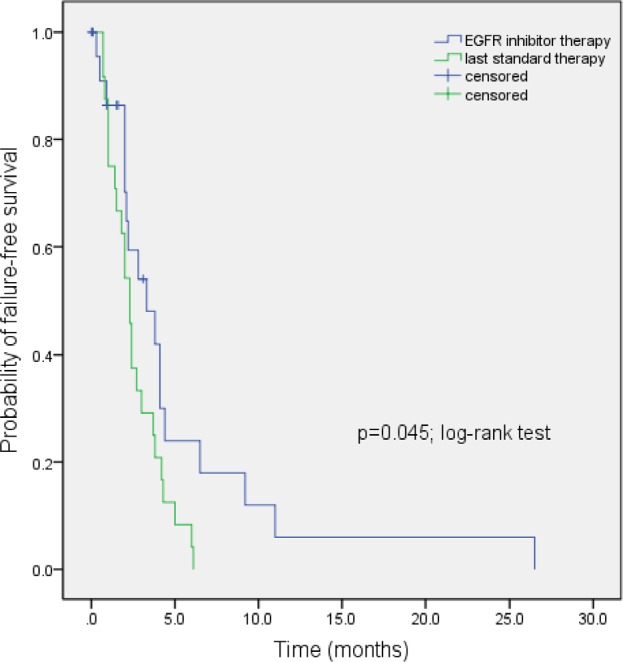
Kaplan-Meier estimates of time to treatment failure in 24 NSCLC patients with *EGFR* wild-type disease treated with EGFR inhibitor-based combination therapies in phase I clinical trial program (3.3 months) vs. time to treatment failure on their last standard therapy (2.3 months; p=0.045; log-rank test)

Of the 24 patients with *EGFR* wild-type disease (Table 3) treated on the same protocols as listed above, 19 were evaluable for response to treatment. Five patients were not evaluable for response, as restaging had not yet occurred at the time of this analysis. Overall, 5 of 19 evaluable patients (26%) had either a PR (n=1, case #12, Table 3; duration = 4.1 months) or SD ≥6 months (n=4; cases #1, 6, 9, and, 10; duration = 26.5 9.2, 6.5, and 11.0 months respectively). All of them were treated on erlotinib/cetuximab/bevacizumab. The median TTF of the 24 patients with *EGFR* wild-type disease treated on EGFR inhibitor-based combinations in phase I clinical trial program is statistically longer (3.3 months; 95% CI, 1.2-5.4 months) compared to median TTF on last standard therapy (2.3 months; 95% CI, 1.8-2.8 months; p=0.045; Figure 3). Four of five patients with SD ≥6 months/PR had not received prior EGFR inhibitors. One patient (case #6, Table 3), who had previously received erlotinib as a single-agent for 6.2 months and had progressed, had SD for 9.2 months on erlotinib/cetuximab/bevacizumab. One of five patients who achieved SD ≥6 months/PR had squamous cell histology (case #1, Table 3; duration of SD=26.5 months). Overall, two evaluable patients with *EGFR* wild-type disease (cases #1 and 14, Table 3) who were treated with EGFR inhibitors had squamous histology.

### Responses in NSCLC patients with squamous cell histology

A total of three evaluable patients treated on EGFR inhibitor-based combinations had squamous cell histology, two of whom had *EGFR* wild-type disease (cases #1 and 14, Table 3), and one of whom had an *EGFR* mutation (case #15, Table 2). One of the two patients with wild-type EGFR (case #1, Table 3) attained SD for 26.5 months on erlotinib/cetuximab/bevacizumab. This patient had received two lines of standard therapy, but was not previously treated with an EGFR inhibitor. TTF on the last standard therapy prior to this study was 2.4 months. A second patient with wild-type *EGFR* (case #14, Table 3) had progressive disease after 2.1 months on erlotinib/cetuximab/bevacizumab. This patient had received only one line of standard therapy (not an EGFR inhibitor) and the TTF was 4.2 months. The third patient was *EGFR* mutation-positive (case #15, Table 2; T847I, unknown sensitivity to EGFR inhibitors) and also had an additional *PIK3CA* mutation (E545K in exon 9); this patient had progressive disease within one month on erlotinib and dasatinib. This patient had received two lines of standard therapy, but not an EGFR inhibitor. TTF on last therapy before referral was 1.0 month.

### Responses in NSCLC patients with other simultaneous mutations

#### PIK3CA

Of the two *EGFR* mutation-positive patients with a simultaneous *PIK3CA* mutation, one patient (case #5, Table 2) with an E542K mutation in exon 9 of the *PIK3CA* gene in addition to two sensitive *EGFR* mutations (L858R and G873E in exon 21), achieved a PR (-55%) for 9+ months on erlotinib/cetuximab/bevacizumab. The other EGFR mutation-positive patient (T847I in exon 21; unknown sensitivity to EGFR inhibitors) with a co-existing E545K mutation in exon 9 of the *PIK3CA* gene (case #15, Table 2), had progressive disease in 0.4 months on erlotinib and dasatinib.

Of the two *EGFR* wild-type NSCLC patients with a E545K mutation in exon 9 of the *PIK3CA* gene, one patient (case #15, Table 3) has ongoing SD at 3+ months on erlotinib/cetuximab/bevacizumab; the other patient (case #23, Table 3) had SD for 3 months on erlotinib/dasatinib.

#### KRAS/TP53

The two *EGFR* wild-type patients with a *KRAS* mutation (cases #20 and 21, Table 3) were not evaluable as they had not reached the post-treatment assessment. None of the 19 evaluable patients with *EGFR* wild-type had a *KRAS* mutation. Of the two *EGFR* wild-type patients with a *TP53* mutation, one patient (case #1, Table 3) had prolonged SD for 26.5 months on erlotinib/cetuximab/bevacizumab; the other patient (case #19, Table 3) had PD after 0.5 months on erlotinib/cetuximab.

### Overall Survival

The median OS of 15 *EGFR* mutation-positive patients treated on EGFR inhibitor-based regimens from the date of start of therapy was 4.7 months (95% CI, 3.5 – 5.9 months). The one year survival rate was 31% (95% CI, 22.3-41.1%). At the time of analysis, 12 of 15 patients were dead. The median OS of 24 *EGFR* wild-type patients treated on the same regimens was 3.8 months (95% CI, 0.6-7.0 months).

## DISCUSSION

The identification of molecular aberrations and selection of therapy to ‘match’ these aberrations is gaining momentum as a preferred treatment approach[14-24]. In the Phase I setting, studies have sought to validate this approach across a range of mutation types, and to support broad genomic testing.

*EGFR* mutations, frequently observed in patients with NSCLC, activate the kinase activity of EGFR, leading to upregulation of downstream survival pathways[25, 26]. In our study, *EGFR* mutations in exons 18-21 were present in 21 of 131 patients (16%) with NSCLC. Despite the dramatic initial responses to single-agent EGFR tyrosine kinase inhibitors (TKIs) in *EGFR* mutation-positive NSCLC patients, 70% of them relapse within one year of initiation of therapy[7, 8, 27]. It has been reported that kinase-independent activity of EGFR prevents autophagic cell death, perhaps accounting for progression of disease despite treatment with tyrosine kinase inhibitors [10]. It is conceivable that combining EGFR kinase inhibitors with EGFR antibody may therefore overcome resistance. Based on preclinical studies, other strategies to augment EGFR inhibition includes use of bortezomib in combination with EGFR kinase inhibitors due to its growth inhibitory and pro-apoptic effects on cancer cell lines[12] and dasatinib in combination with EGFR kinase inhibitor as *in vitro* data from NSCLC cell lines demonstrates that Src inhibition may enhance the antitumor activity of EGFR inhibition in the presence of *EGFR* mutations[11].

In our study, eight of 34 evaluable patients (23%) treated with EGFR inhibitor-based combinations achieved SD ≥6 months/PR. Those individuals include EGFR mutation-positive patients with a *de novo* resistant mutation (n=1); *EGFR* mutation-positive (sensitive mutation) and prior secondary resistance to EGFR inhibitors (n=2); and, *EGFR* wild-type disease (n=5; including a patient with squamous cell histology).

Of the 15 *EGFR* mutation-positive NSCLC patients treated on EGFR inhibitors, three (20%) had SD ≥6 months/PR (cases #2, 5 and 10, Table 2). One patient with a known *EGFR*-resistant mutation (insertion in exon 20; case #2, Table 2) has an ongoing PR (33% decrease) at 13+ months on therapy with erlotinib and the EGFR antibody cetuximab. Indeed, there is preclinical data demonstrating superiority when EGFR kinase inhibitors are combined with EGFR antibody in regard to antitumor effect[28, 29]

A second patient (case #5, Table 2) with two known *EGFR*-sensitive mutations (L858R and G873E in exon 21) attained a PR (55% decrease) for 9+ months on erlotinib/cetuximab/bevacizumab. Interestingly, this patient also had a *PIK3CA* mutation (E542K in exon 9) in the downstream signaling pathway which is a known resistant mechanism to EGFR inhibition[30]. The *PIK3CA* mutation may explain why the patient had previously developed secondary resistance to single-agent erlotinib. Preclinical studies have demonstrated antitumor activity with PI3K/mTOR inhibitor combinations in gefitinib-resistant *PIK3CA*-mutant NSCLC cell lines[31]. Clinical trials with dual blockade of PI3K and mTOR are underway[32, 33]. The response seen in this patient on erlotinib/cetuximab/bevacizumab may be due to synergistic effect of simultaneous EGFR and vascular endothelial growth factor (VEGF) inhibition, as demonstrated in preclinical models, including EGFR inhibitor-resistant cell lines[34] and in patients with metastatic colorectal cancer[35], and NSCLC[36, 37]. A third patient (case #10, Table 2) with a known *EGFR*-sensitive mutation (L858R), with previous response to single agent erlotinib and subsequent resistance, has ongoing SD (26% decrease) for 10+ months on erlotinib and bortezomib. There is preclinical evidence that dual blockade of proteasome activity and EGFR function by combining bortezomib plus cetuximab has synergistic antitumor activity[12]. Patients treated with erlotinib may also benefit from re-treatment[38], so this could also explain the observation of prolonged stable disease, at least in part. However, the TTF on prior erlotinib was 5.5 months, while the current TTF is 10+ months, suggesting that the longer current TTF could not be due to erlotinib alone. These observations indicate that combining treatment with drugs that target different signaling pathways may help patients who had progressed on single-agent targeted therapy after a period of initial response.

Unexpectedly, patients with *EGFR* wild-type disease were also noted to have salutary effects on EGFR inhibitor-based combination therapies. Five of 19 evaluable NSCLC patients with wild-type EGFR treated on the same EGFR inhibitor-based regimens attained SD ≥6 months/PR. The median TTF on EGFR inhibitor-based regimen (3.3 months) was significantly longer than the median TTF on their last standard therapy (2.3 months; p=0.045; Figure 3). These data suggest that EGFR-based combinations can be active in patients with wild-type disease[39, 40]. All these patients received erlotinib/cetuximab/bevacizumab. Previously, modest antitumor activity has been reported in 4 of 13 NSCLC patients with wild-type EGFR (31%) on gefitinib and cetuximab[41].

We also noted anecdotal activity in patients with squamous cell histology. Of the three evaluable patients with squamous cell histology who were treated with EGFR inhibitor-based regimens, two patients had *EGFR* wild-type disease (cases #1 and 14, Table 3) and one patient was *EGFR*-mutant (case #15, Table 2). One patient (*TP53*-mutation positive; case #1, Table 3) with *EGFR* wild-type, squamous cell histology achieved SD for 26.5 months on erlotinib/cetuximab/bevacizumab after progression on two prior therapies. Limited data exists regarding patients with squamous cell histology treated with EGFR inhibitors because these patients typically have *EGFR* wild-type disease[42]. In one study [43], 121 patients with squamous cell carcinoma of the lung were treated with single-agent erlotinib. Thirty seven of 69 evaluable patients achieved PR/SD; however, the duration of response or molecular aberrations was unknown. In another study[44], 1,125 NSCLC patients including 190 patients with squamous cell carcinoma were randomized to either chemotherapy alone or to cetuximab plus chemotherapy. A survival advantage was observed in 557 NSCLC patients treated with cetuximab plus chemotherapy; however, information on which of these responders had squamous cell histology was not reported. Finally, one responsive patient was noted to have a *TP53* mutation. Recent retrospective data analysis posits longer progression-free survival for *TP53*-mutant patients on bevacizumab-based regimens[45].

The activity noted in these subtypes of NSCLC is hypothesis-generating; however, sample size is a significant limitation of this study. The heterogeneity of combination treatments and the retrospective nature of the analysis must also be considered. Therefore, any interpretation of these results needs to be approached with caution. Furthermore, the antitumor activity seen in two patients with *EGFR*-mutant NSCLC (cases #5 and 10, Table 2), who had progressed on prior treatment with erlotinib after initial response, may be due to the re-treatment effect that occurs in patients with EGFR mutant disease with reintroduction of an EGFR tyrosine kinase inhibitor after a drug holiday[38]. However, in the latter case (case #10, Table 2), the TTF is ongoing and at least double the TTF on prior EGFR inhibitor therapy.

In conclusion, this study demonstrated that treatment with EGFR inhibitor-based combination therapies was associated with SD ≥6 months/PR in subtypes of heavily pretreated advanced NSCLC not traditionally associated with response to EGFR inhibitors, including 1 of 2 patients with a *de novo EGFR*-resistant mutation; 2 of 12 patients (17%) with an *EGFR*-sensitive mutation and secondary *EGFR* resistance after a period of initial response; 5 of 19 evaluable patients (26%) with *EGFR* wild-type disease; and, 1 of 3 evaluable patients (25%) with squamous cell carcinoma. Further exploration of rational EGFR inhibitor combinations in a broad range of patients with NSCLC may be warranted.

## METHODS

### Patients

We investigated the *EGFR* mutation status of 131 consecutive patients with NSCLC referred to the Department of Investigational Cancer Therapeutics (Phase I Clinical Trials Program) at The University of Texas MD Anderson Cancer Center (MDACC) beginning January 1, 2009. The study and all treatments were conducted in accordance with the guidelines of the MD Anderson Institutional Review Board.

### Tissue samples and mutation analyses

*EGFR* mutations were investigated in archival formalin-fixed, paraffin-embedded tissue blocks or material from fine needle aspiration biopsy obtained from diagnostic and/or therapeutic procedures. All histologies were centrally reviewed at MDACC. *EGFR* mutation testing was done in the Clinical Laboratory Improvement Amendment–certified Molecular Diagnostic Laboratory within the Division of Pathology and Laboratory Medicine at MDACC.

DNA was isolated from formalin-fixed, paraffin-embedded tissue by using a QIAmp DNA Minikit (Qiagen Inc., Valencia, CA) according to the manufacturer's instructions. *EGFR* exons 18-21 sequence were analyzed in both sense and antisense directions for the presence of mutations using nested PCR followed by direct sequencing of the nested PCR amplicons. The nested-PCR was done using the primers and annealing conditions as described by Lynch et al[3]. The nested PCR amplicons were purified using the Qiagen QIAquick PCR Purification Kit, followed by cycle-sequencing using BigDye Terminator Kit v1.1 (ABI, Foster City, CA) on ABI Prism 3130 Genetic Analyzer, according to manufacturer's instructions. Whenever possible, in addition to *EGFR*, we tested for other mutations such as *PIK3CA* (codons 532 to 554 in exon 9 and codons 1011 to 1062 in exon 20), *KRAS* (codons 12, 13, and 61) and *TP53* (exons 4 to 9).

### Treatment and evaluation

EGFR inhibitors included the small molecule inhibitor erlotinib and the antibody cetuximab. Rationale for the trials was based on preclinical work demonstrating synergistic or additive effects and/or clinical work suggesting complementary pathway inhibition. The trials included erlotinib/cetuximab/bevacizumab (ClinicalTrials.gov identifier: NCT00543504); erlotinib/cetuximab (ClinicalTrials.gov identifier: NCT00895362); erlotinib/bortezomib (ClinicalTrials.gov identifier: NCT00895687); erlotinib/dasatinib (ClinicalTrials.gov identifier: NCT00895128); and cetuximab/sirolimus (ClinicalTrials.gov identifier: NCT00940381). Treatment was chosen based on trial availability and physician and patient preference. Treatment continued until disease progression or unacceptable toxicity occurred. Treatment was carried out according to the specific requirements of the treatment protocols selected. Assessments, including history, physical examination, and laboratory evaluations, were done as specified in each protocol, typically before the initiation of therapy, weekly during the first cycle, and then, at a minimum, at the beginning of each new treatment cycle.

### Response assessment

Efficacy was assessed from computed tomography (CT) scans and/or magnetic resonance imaging (MRI) and/or positron emission tomography (PET) scan at baseline before treatment initiation and then every 2-3 cycles (6–12 weeks), depending on the protocol. All radiographs were read in the Department of Radiology at MDACC and reviewed in the Department of Investigational Cancer Therapeutics tumor measurement clinic. Responses were categorized per Response Evaluation Criteria in Solid Tumors (RECIST) 1.0 [46] criteria and were reported as best response. Complete response (CR) was defined as the disappearance of all measurable and non-measurable disease; partial response (PR) was defined as at least a 30% decrease in the sum of the longest diameter of measurable target lesions; progressive disease (PD) was defined as at least a 20% increase in the sum of the longest diameter of measurable target lesions, or unequivocal progression of a non-target lesion, or the appearance of a new lesion; and stable disease (SD) was defined as neither sufficient shrinkage to qualify for PR nor sufficient increase to qualify for PD. A waterfall plot was used to illustrate the anti-tumor activity observed in patients evaluable for response.

### Statistical analysis

Patient characteristics, including demographics, *EGFR* mutation status and prior treatment with an EGFR inhibitor were summarized using frequencies and percentages. Time to treatment failure (TTF) was defined as the time interval between the start of therapy and the date of disease progression or death, whichever occurred first. Patients who were alive and had not failed treatment were censored at the time of their last follow-up. The Kaplan-Meier method [47] was used to estimate TTF and log-rank tests [48] were performed to compare subgroups of patients. All tests were two-sided, and P <0.05 was considered statistically significant. All statistical analyses were carried out using SPSS (version 19.0; SPSS, Chicago, IL, USA).
